# Efficient Exact Maximum a Posteriori Computation for Bayesian SNP Genotyping in Polyploids

**DOI:** 10.1371/journal.pone.0030906

**Published:** 2012-02-17

**Authors:** Oliver Serang, Marcelo Mollinari, Antonio Augusto Franco Garcia

**Affiliations:** 1 Department of Neurobiology, Harvard Medical School, Boston, Massachusetts, United States of America; 2 Department of Pathology, Children's Hospital Boston, Boston, Massachusetts, United States of America; 3 Department of Genetics, University of São Paulo/ESALQ, Piracicaba, São Paulo, Brazil; University of East Piedmont, Italy

## Abstract

The problem of genotyping polyploids is extremely important for the creation of genetic maps and assembly of complex plant genomes. Despite its significance, polyploid genotyping still remains largely unsolved and suffers from a lack of statistical formality. In this paper a graphical Bayesian model for SNP genotyping data is introduced. This model can infer genotypes even when the ploidy of the population is unknown. We also introduce an algorithm for finding the exact maximum a posteriori genotype configuration with this model. This algorithm is implemented in a freely available web-based software package SuperMASSA. We demonstrate the utility, efficiency, and flexibility of the model and algorithm by applying them to two different platforms, each of which is applied to a polyploid data set: Illumina GoldenGate data from potato and Sequenom MassARRAY data from sugarcane. Our method achieves state-of-the-art performance on both data sets and can be trivially adapted to use models that utilize prior information about any platform or species.

## Introduction

Most agriculturally important plant species, such as potato, sugarcane, coffee, cotton and alfalfa, are polyploids. In fact, about half of the natural flowering plant species are polyploids [1]. Despite their importance, our understanding of these species does not fully benefit from marker technology. Molecular markers are widely used for diploid species and can be very useful for building linkage maps [2], finding genomic regions associated with variation in quantitative traits (or QTL) [3], studying the genetic architecture of quantitative traits [4], and assembling genome sequences [5].

Accurate genotyping of polyploids (even for largely uncharacterized species or in cases when the ploidy is unknown) is a missing keystone in genetics that must be solved in order to utilize the approaches that have marked a revolution in biology over the past hundred years. Accurate genotypes are necessary to understand the genetic mechanisms and specific loci that determine phenotypes via QTL mapping and association studies. These genotypes are also necessary for the creation of linkage maps, which are exceedingly useful in developing a greater understanding of genome evolution. These linkage maps will be essential for the assembly of complex polyploid genomes.

The current approach used for several genetic studies on polyploids, especially for linkage mapping, is based on marker loci with only a single copy (simplex) in one of the parents and a nulliplex in the other, in 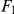 populations obtained from the cross of non-inbred parents. Markers such as AFLP and SSR ( *i.e.* microsatelites) are then scored as presence or absence of bands [6]–[8] and behave like dominant markers. For sugarcane, most available linkage maps are based on markers segregating in 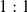 (single dose in one parent) or 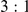 patterns (single dose in both parents) [9]. Even if complex statistical methods are applied to obtain integrated maps that combine information from markers with both patterns simultaneously [10], [11], the available maps are based on a small sample of the genome, since markers with higher doses are normally not included; therefore, they are not well saturated and informative for genome assembly [12]. For QTL studies in sugarcane, the situation is similar. Statistical models developed for backcrosses are used for simplex
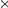
nulliplex configurations with available software that was developed for diploids [13]. Since the ploidy level could be related with gene expression [14], these approaches need to be modified to incorporate allele dosage using more efficient marker systems.

Nowadays, new technologies such as Illumina GoldenGate™ [15] and Sequenom iPLEX MassARRAY® [16] allow researchers to generate high-throughput genotyping data from SNPs. These data usually contain two signals for each SNP locus, each one corresponding to an intensity recorded for one of the two possible alleles. The expected value of each signal intensity is proportional to the corresponding allele dosage [16], [17], and therefore SNPs are the marker of choice for genetic studies in polyploids. They are more informative than presence/absence markers, and should allow a better coverage of the genome and the development of more realistic models for linkage studies, QTL and association mapping, among other applications.

In order to explore the full potential of such technologies, a first required step is the development of statistical methods for SNP genotype calling, *i.e.* inferring the (discrete) genotype of each individual for each locus, identifying the number of copies of each allele. For diploids, including humans, a number of methods are already available [18]. This is not the case for polyploids. Methods for polyploid genotyping need to be able to deal not only with multiple copies of the alleles, but also with some complex problems such as aneuploidy and unknown ploidy, which can be present for some species.

Voorrips *et al.* [19] presented an approach based on mixture models for genotype calling in autotetraploids, in a similar way as done by [20] in diploids. Based on the (transformed) allele signal ratio (ratio of one signal peak to the total), they fitted a mixture of five normal distributions, each one corresponding to one genotype class (from zero to four copies of the allele). They compared several models and were also able to test for Hardy-Weinberg equilibrium in a potato panel with 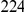 tetraploid potato varieties. Their model could be expanded for allowing the inclusion of more classes in the mixture in order to be useful for other autopolyploids; however, in certain situations the ploidy (and hence the number of classes) is unknown and need to be estimated. Also, crosses with distinct ploidies and parents may result in similar segregation patterns, making the selection of the best model a complicated task. This is the case for sugarcane, which is a very complex polyploid and aneuploid species. Genotype calling in sugarcane is extremely difficult, especially if commercial varieties are used, since they are interspecific hybrids between domesticated and wild relatives [21].

Here we present a graphical Bayesian model for SNP genotyping calling. Our graphical Bayesian method can infer genotypes even when the ploidy of the population is unknown. At the core of Bayesian thinking is the notion of modeling processes forwards rather than trying to model their inverse. Generally, a great deal of prior knowledge is available regarding the way any process behaves running forwards; when the process is modeled generatively ( *i.e.* running forwards), this prior knowledge can be exploited to improve the fidelity with which it describes the process. In graphical models prior knowledge regarding independence and conditional independence of variables can be visualized in the structure of the graph. The highly connected subunits of the graph can be considered with modularity; that is, a subunit can be easily interchanged with another. This modularity is what allows our model to work with populations in Hardy-Weinberg equilibrium, the progeny of an 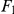 cross, or any population with a known theoretical distribution of genotypes. This modularity results in a model and inference procedure that are compatible with any theoretical distribution of genotypes in the population. There are many other ways that our model, and similarly motivated models, can be easily changed and improved because of their modularity and generality.

We also introduce an algorithm for finding the exact maximum a posteriori (MAP) genotype configuration with this model. This algorithm is implemented in a freely available software package named SuperMASSA. We demonstrate the utility, efficiency, and flexibility of the model and algorithm by applying them to data from two polyploids processed with two different platforms: potato [19] using Illumina GoldenGateTM assay [15] and sugarcane using Sequenom iPLEX MassARRAY® [16].

## Materials and Methods

### Data

#### Potato

**Figure 1:**
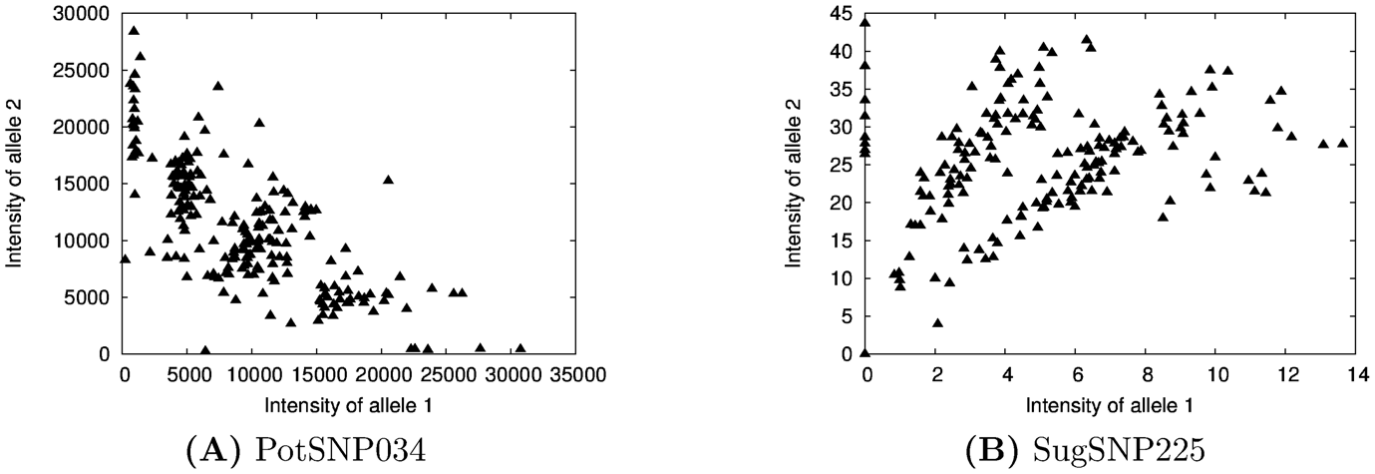
Raw data. The scatter plot of allele intensities for PotSNP034 (**A**), tetraploid, and SugSNP225 (**B**), which has an unknown ploidy.

An autotetraploid potato collection was used, comprising 384 SNPs scored in a panel of 224 individuals using the Illumina GoldenGateTM assay, as described in [22] and [19]. This data set is distributed along with the free R package fitTetra [23], under the GNU General Public License. To exemplify the results obtained using the mixture model, [19] chose three loci, PotSNP016, PotSNP034 (Figure 1) and PotSNP192. However, for loci PotSNP192, they noted that the Illumina GoldenGate assay produced significantly different signal strengths for the alleles, resulting in skewed clusters. Thus, the intensity ratio between those alleles can not be easily used to infer genotypes. Since our model assumes the signal strength of each allele is proportional to the dosage (and that the proportionality constant for both alleles is similar), we used only PotSNP016 and PotSNP034 to exemplify our method. For this data set, we use the same model of the genotype distribution as [19] ( *i.e.* Hardy-Weinberg). Moreover, since we know the ploidy for both the diploid and tetraploid potatoes, we can check if the ploidy estimated by our model matches the actual one. These two SNPs were also scored in 64 diploid potato varieties that were used for a visual check of the goodness of fit. We also analyze the diploid individuals using PotSNP016 and PotSNP034.

#### Sugarcane

A sugarcane mapping population derived from a cross between two commercial varieties (IACSP 95-3018
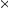
IACSP 93-3046) was used. It was comprised of 180 individuals scored for 241 SNPs using the Sequenom iPLEX MassARRAY® technology [16]. This assay is based on allele-specific primer extension with a mass-modified terminator [24]. The DNA products of this reaction are analyzed by a MALDI-TOF mass spectrometer and each polymorphic region of interest is detected by a mass of the allele-specific primer [25]. Both parents were also scored 12 times for each SNP. If the ionization efficiency is similar for both alleles, the intensities produced by mass spectrometry are proportional to abundance (with very similar proportionality constant if run in the same sample prep); therefore, the if the amplification of both alleles is similar, the skew is minimal. We observe much less skew in the sugarcane data set compared to the potato data set.

Modern sugarcane varieties have highly polyploid and aneuploid genomes, with ploidy levels ranging from 5 to 16 [26], [27]. Therefore, unless there is strong cytological information for a marker, it is important to also estimate the ploidy. Since we want to test our model and do not have a reference point for sugarcane (such as the known diploids or tetraploid potato varieties), and also because sugarcane meiosis frequently result in deviations from the expected Mendelian segregation ratios [26]–[28], we used a blind method to curate the data and evaluate SuperMASSA.

First, all sugarcane loci were curated by eye using several criteria. For each locus, an expert looked at raw scatter plots as shown in Figure 1 and assessed the following: *i*) the overall quality; *ii*) the number of clusters; and *iii*) the expected ploidy level based on parental data. This resulted in 27 SNPs that were easily classified by eye. SuperMASSA was used to predict the ploidy and number of clusters for each of these 27 loci and three of them (the three judged to be of the highest quality) are used to show the results of our model.

It is important to note that in this blind validation experiment, SuperMASSA was not used to curate the data and the model behind SuperMASSA was not changed after observing and curating the data.

### Probabilistic Graphical Model

We use a Bayesian approach to model the probability of the observed data given the ploidy and all genotypes. By modeling the generative process ( *i.e.* the process by which the data is produced assuming we know the ploidy and genotypes of all individuals), we can build the model from realistic assumptions for the data. Using the model, we then perform inference (described in the Probabilistic Inference section) to effectively enumerate all possible ploidies and genotypes for individuals in the population, and choose the configuration that maximizes the posterior probability of the model. This configuration is known as the *maximum a posterior* (MAP) and is guaranteed to result in the highest possible probability.

**Figure 2:**
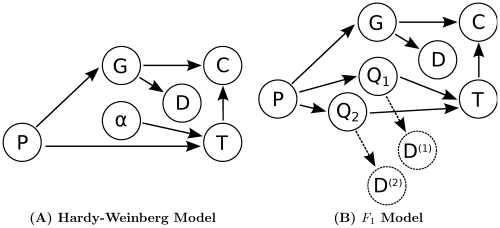
A Graphical View of SNP Genotyping. Two models for SNP genotyping are presented. Variables are shown as nodes and solid arrows depict dependencies between variables. The observed data 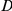 depend on the genotypes of all individuals 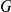. In both models the distribution of genotypes 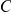 is determined by the genotype configuration 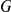. Also, in both models the probability of a genotype distribution depends on 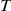, the distribution of genotypes in the population. Furthermore, both models use the same method to compute the probability of the data given the genotype configuration. Lastly, the possible genotypes depend on the ploidy 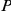. (**A**) In the Hardy-Weinberg model, the distribution of genotypes in the population is determined by one of the allele frequencies 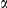. (**B**) In the 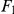 model, the distribution of genotypes in the population depends on the parent genotypes 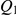 and 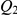. The dashed arrows and nodes (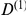 and 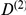) depict optional dependencies and variables; these variables and dependencies exist only when data from the parents is included.

In Figure 2 we present two probabilistic directed graphical models of the SNP genotyping process for a single locus: a Hardy-Weinberg model and an 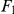 model. These models represent dependencies using directed edges. Both models share similar motivation and notation; the few differences arise from different models of the distribution of genotypes in the population. We first present the shared model components and then present the details specific to each model.

#### Hardy-Weinberg and 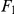 Model Similarities

For both models, the “genotype configuration” 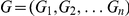 is the collection of genotype assignments for all individuals in the data set. Because the ploidy, denoted 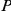, determines the possible set of genotype outcomes, the genotype configuration depends on the ploidy 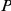. Denote the set of possible genotype outcomes for a given ploidy as 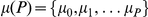. For example, for a diploid locus 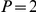 and the set of possible genotypes is 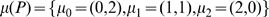. Both models use a uniform prior on the ploidy 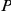; it should be noted that for the data we analyzed, the influence of any weak priors is negligible because of a pronounced drop in suboptimal posteriors relative to the MAP configuration.

The observed data 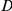 is composed of a collection of data points 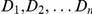, each of which comprises an 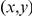 intensity pair and an individual 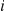 that gave the sample producing the 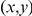 pair. We assume that each data point depends only on the individual that produced it; therefore, the likelihood of any genotype configuration 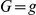 can be written as a product over individuals:
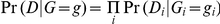
For some 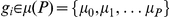, we model the likelihood proportional to 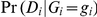 using a normal distribution with unknown standard deviation 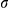:
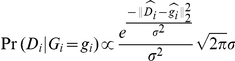
where the operator 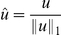 is used to perform 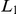 normalization on 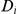 and 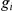. This likelihood effectively uses the expected angles of each genotype and penalizes individuals deviant from the genotype of the expected angle. For this reason, “skewed” data, where the intensities measured by allele 1 and allele 2 use very different constants of proportionality with their respective dosages, cannot be modeled without including a latent variable for the skew. Sigma is given a uniform prior and inference is performed in a manner similar to inference over all ploidy.

For any genotype configuration 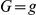, both models also compute 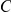, the distribution of possible genotypes. 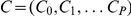, where 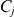 equals 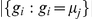, the number of individuals assigned to genotype 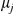. The probability of any distribution 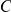 is modeled using the theoretical distribution of genotypes 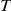. Given the theoretical genotype frequencies for the population 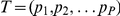 where 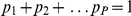, the probability of observing any genotype distribution 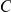 is multinomial:
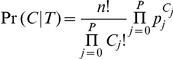
Both the Hardy-Weinberg and 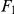 models allow for individuals with replicate data points. If all individuals have the same number of replicate data points, then the MAP configuration is guaranteed to be found (as shown in the Supplement S1).

#### Hardy-Weinberg Model

Figure 2A depicts the dependencies of the Hardy-Weinberg model. In the Hardy-Weinberg model, the theoretical distribution of genotypes is modeled using a binomial distribution. Given 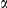, the allele frequency of the first allele (in the ordered pair), the probability of any genotype 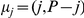 is 
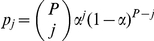
. The parameter 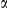 is modeled using a uniform prior. To perform grid search, we discretize 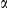 into the range 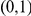 with a resolution of 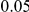.

#### 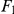 Model

Figure 2B depicts the dependencies of the 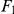 model. In the 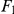 model, the theoretical distribution of genotypes is modeled using hypergeometric distributions for the gametes (it is important to note that any model could be trivially applied instead). Denote 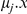 to be the dosage for the first allele in the ordered pair and 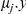 to be the dosage of the second allele in the pair. Given parents 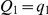 and 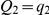, both which have values in 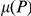, the probability of observing gamete 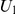 from 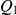 (without loss of generality) is
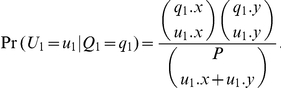
Therefore, the probability of observing offspring 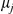 is
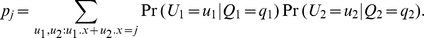
In the 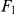 model, the parent genotypes 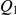 and 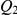 depend on the ploidy since the outcomes of both must be in 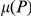. We model the prior probability as uniform for the number of unique outcomes: 
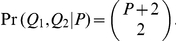


In Figure 2B dashed nodes and arrows represent variables and dependencies that exist only when data from the parents is included. The probability of these parameters can be modeled as conditionally independent, just like 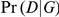:
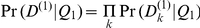
When parental data is used, the parents are distinct and so the number of unique parental combinations becomes 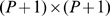; therefore, when parental data is available, the prior probability on parental configurations becomes uniform over these 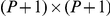 distinguishable outcomes.

#### Generalized Population Model

The inference procedure described does not make any special use of the type of parameters that determine 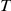; therefore, given the parameters 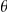 that determine 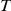 (and do not depend on 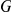, 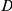, or 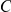), our inference method will find the MAP genotype configuration. This illustrates that both the Hardy-Weinberg and 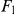 models are specific instances of a general model (where 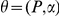 and 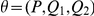, respectively). 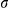 is searched in a similar manner, but since we use a uniform prior, we search all parameter configurations for a given 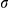 and omit 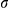 from 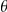 for simplicity (this strategy also allows us to cache the table of likelihoods for a given 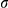). When parental data is included in the 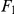 model, it can be modeled by setting the prior probability (that is, the probability including available parent data but excluding data from progeny) to
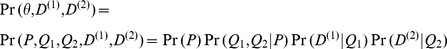



We define the “generalized population model” as the model defined using 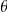. For each 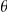 we will compute the MAP genotype configuration 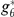; using the prior probability of 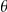, we can enumerate the possible outcomes of 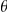 and compute both the genotype configuration and parameters 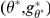 that jointly maximize the posterior probability for these parameters. Using this approach we can also approximate 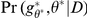, the posterior belief that the MAP parameter and genotype configuration is correct.

### Identifiability

Before inference is performed, it is necessary to demonstrate that the parameters 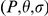 can be inferred with a sufficient amount of data ( *i.e.* they are “identifiable”). By the law of large numbers, the densities of the genotypes and allele intensities converge to the density expected from the parameters 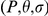 as 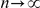; therefore, with enough individuals, the exact distribution of genotypes and allele intensities is known. In order to prove that the parameters are identifiable, we must demonstrate that 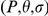 can be computed from this density 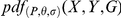 ( *i.e.* that 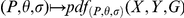 is one-to-one). It is sufficient here to prove that no two non-identical pair of parameters 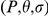 can yield the same density.

By assumption, our model considers data which is a weighted sum of Gaussians (one for each genotype), each with a mean 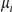 at the expected slope for the two allele intensities. Algabraically, for two densities to be equal, the two equivalent sums of shifted Gaussians, each of the form 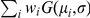, must use identical sets 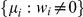 (when 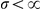). Furthermore, the corresponding weights 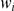 must be equal for Gaussians shifted by the same 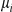. Together, these statements require that identical densities must be created by sets of parameters with identical angles 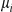 for all possible genotypes (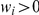). This requires that all genotypes have an equal dosage to ploidy ratio for each possible genotype.

If this set of 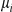 contains more than one possible genotype, then the difference between the two dosages increases for the larger ploidy (because the ploidy, the denominator in both slopes, has increased, but the slopes remains constant). Because these dosages are necessarily integers, then the difference must increase by at least one, indicating a new genotype class with expected slope between the other two. Therefore, to have the same set of 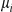, the larger ploidy has a possible genotype class not possible with the smaller ploidy, and this genotype class is not possible with the smaller ploidy. Thus, the larger ploidy must assign a weight 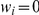 to that new genotype class.

However, both models considered (Hardy-Weinberg and 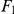) create unimodal (or flat) weight distributions. For this reason, they cannot create sequential weights that are nonzero, zero and then nonzero again. Furthermore, given the ploidy, the weights (or expected frequencies) are sufficient to estimate 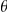. Therefore, if more than one possible genotype exists, the parameters are identifiable (the lowest ploidy that could produce the desired angles is the only one possible). When only one possible genotype exists, the ploidy cannot be estimated (it could be any multiple of a ploidy that produces the correct angle). In this case, we use an Occam's razor approach by placing a decreasing prior on the ploidy 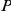.

### Probabilistic Inference

In order to perform inference on the generalized population model described in the Probabilistic Graphical Model section, we introduce three approaches: a greedy approach (maximum likelihood), an exact approach (MAP) via dynamic programming, and a substantially more efficient exact approach (also MAP). For all inference methods, assume 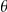 is known. The best greedy genotype configuration and 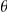 can be chosen by enumerating all outcomes of 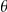 and selecting the one with highest posterior.

Graphically, it is trivial to demonstrate why MAP inference is difficult. Consider 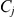, a single bin in the distribution 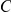; it has incoming edges from all individuals' genotypes 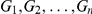. Thus, in the the moral graph (in which all nodes with a common successor are joined by an undirected edge), an edge joins each pair of nodes 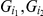, resulting in a clique of size 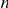. The treewidth [29] of a graph containing an 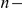
clique is at least 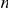, so standard inference methods ( *e.g.* naive enumeration or junction tree inference [30], [31]) will require number of steps exponential in 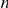 at least; for problems of the size we consider (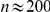), a runtime exponential in 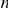 is infeasible.

#### Greedy Inference

Rather than jointly consider all genotype assignments, the greedy approach approximates 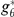 by using maximum likelihood estimation. The likelihood considers only 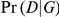. Because of conditional independence of data given the genotype configuration, the maximum likelihood genotype configuration is defined:
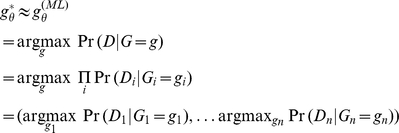
The greedy estimate can independently compute the most likely genotype of each 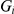 individually, effectively ignoring their combinatorial joint dependencies.

For each 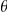, the maximum likelihood genotype configuration 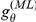 can be evaluated by computing the joint probability with the data. Denote the distribution resulting from a given genotype configuration 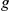 as 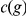. Then the joint probability given 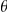 can be written as follows:
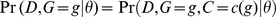
(1)

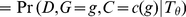
(2)

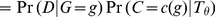
(3)Using the equation 3, the configuration with the highest joint posterior
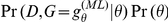
can be found by enumerating outcomes of 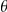.

#### Exact Inference

The combinatorial dependencies between genotypes in different individuals must be recognized in order to compute the MAP genotype configuration. It is tempting to approximate these dependencies with a mixture model. A mixture model approach treats all 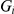 as independent draws from the distribution 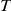; however, a mixture model rewards configurations assigning all individuals the most probable genotype in 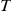. In reality, such a configuration is extremely improbable because there is only one series of genotype assignments that result in this outcome. On the other hand, if 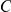 is chosen so that not all individuals are assigned the most probable genotype in 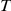, the multinomial probability may be larger because there are many genotype configurations that could lead to 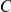 (compared to the single configuration that yields the most probable genotypes). Modeling this dependency between all individuals, although computationally challenging, is extremely important.

**Figure 3:**
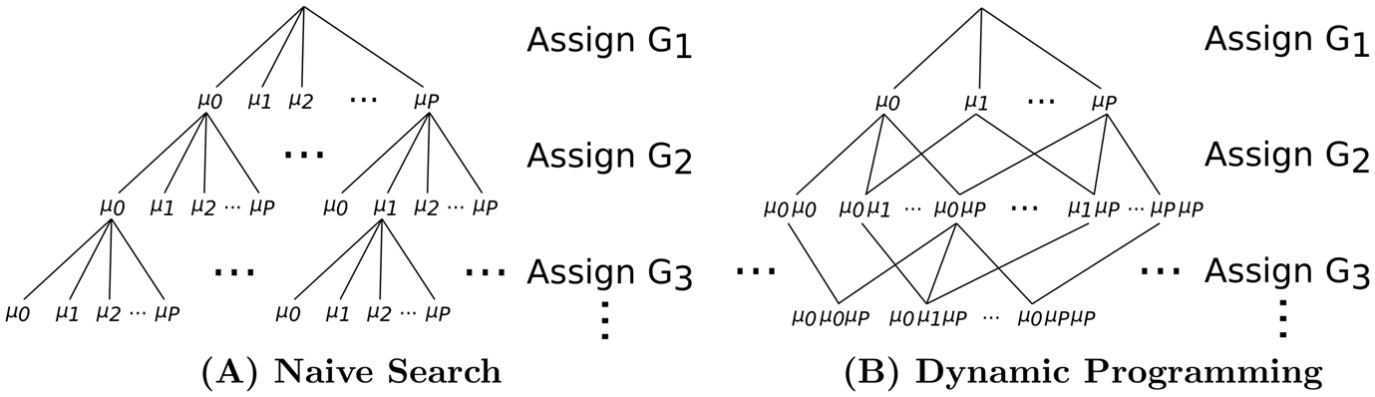
Illustration of Exact Inference. Exact MAP computation can be performed by enumerating all possible genotype configurations. Because each individual's genotype is among 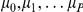, searching through genotype configurations can be viewed as a tree in which each individual genotype assignment branches into 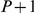 separate outcomes. (**A**) A naive search progresses downward through the tree and chooses the series of genotype assignments that lead to the highest posterior probability. A naive branch and bound method derived from this tree bounds genotype configurations for which the prefix determines that all subsequent paths are poor. (**B**) A multinomial graph ( *i.e.* the subset graph of the power-set of G) merges outcomes that result in the same genotype counts 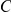. Multiple paths (from the top) can lead to any given set of genotype counts; therefore, dynamic programming is used. Given the layer above, each node can compute the most likely path from the top that leads to it. Once the most likely path and score are computed for each node in a layer, the next layer can progress. At the bottom layer, the node with the highest combined likelihood 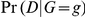 (computed via dynamic programming) and 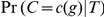 (the same for any path terminating at the node) maximizes the posterior probability. As in the naive tree, once all lower adjacent nodes in a subtree are provably suboptimal, then the subtree can be bounded. The dynamic programming approach is substantially more time space-efficient than the naive approach.

In the simplest approach, all possible genotype configurations can be enumerated naively in exponential time, resulting in the tree shown in Figure 3A. Although it is infeasible to think of enumerating the entire tree, it may be possible to ignore subtrees that cannot lead to an optimum, substantially reducing the search space.

Consider individuals in an arbitrary order with some genotypes assigned: Let 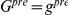 denote 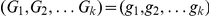 for 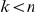 and 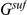 denote the unassigned genotypes 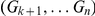. We refer to the assigned genotypes 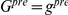 as a “prefix” genotype configuration and the unknown 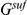 as a “suffix”. Given a prefix genotype configuration, it is possible to bound the joint probability of all configurations with this prefix by bounding the likelihood for the remaining configurations:
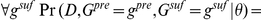


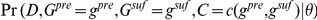


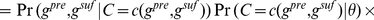


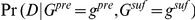


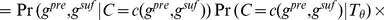


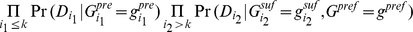


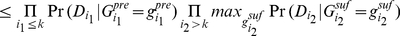
(4)


Given a genotype and parameter configuration 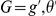, any configuration including the prefix satisfying the following inequality is suboptimal:
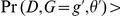


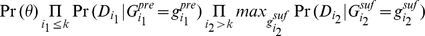
The prefixes correspond to paths from the top of the tree in Figure 3A; prefixes that are shown to be suboptimal can be “bound,” meaning that they are not branched and searched further down. The second product may be cached for all 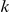 for a speedup of 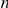. It is worth noting that this second product must be included, because the likelihood constant on 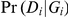 is unknown and so we cannot guarantee that 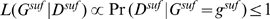. With all of the branch and bound approaches, the initial values 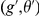 can be computed using the greedy maximum likelihood approach and then improved as more probable configurations are found.

A more sophisticated dynamic programming approach (shown in Figure 3B) merges nodes of equal depth that produce identical distribution prefixes
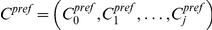
and the number of individuals with each genotype in the genotype prefix. Because 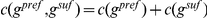, then if two prefixes 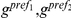 produce the same distribution prefixes 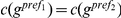, the suffixes satisfying 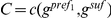 are the same as the suffixes satisfying 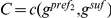. For this reason, other than the prefix likelihoods 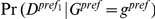 and 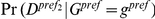, all other values in equations 4 will be the same; therefore, all prefixes producing the same prefix distribution can be grouped together, using the greatest prefix likelihood and corresponding prefix path. These grouped nodes can be added in batches for each depth to produce a “layer;” by induction the best path to each node in a layer includes the best path to the nodes in the layer above. The same bound from the naive tree is used, but subproblems that are identical are grouped and solved together to avoid redundant computation and storage.

#### Efficient Exact Inference

There are a number of reasons that the naive and dynamic programming branch and bound methods are inefficient. First, the number of nodes visited in these trees may be as much as 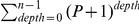 and 
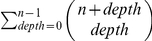
, both of which are exponential in 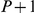. This number of nodes determines the time and (if implemented in a manner that emphasizes runtime efficiency), the space required. Secondly, the suffix path is unconstrained; given 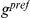, there is no restriction on 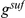, and so the bound must use the maximum likelihood for the remaining 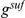 likelihood. Most importantly, the bound in equation 4 is very conservative; in order to bound a subtree with prefix 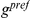, the overall likelihood of all subsequent trees must be less than the product of the overall likelihood and multinomial multiplier 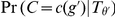 for a full configuration 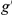. Because even the largest multinomial probability 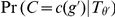 is usually very small, the bound is extremely conservative. It is not feasible to use either the naive or dynamic programming branch and bound methods on the presented data.

For these reasons, we introduce a novel geometric branch and bound method; this method has several advantages. First, when the number of individuals is substantially larger than the ploidy (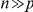), the worst-case tree produced by our method is several orders of magnitude smaller (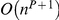 rather than 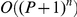). Secondly, our geometric method allows us to substantially constrain valid suffix configurations. Lastly, our method makes use of the multinomial probability in the bound; this multinomial probability is very influential in selecting the optimum (especially when the optimal 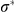 is not very close to zero). Our geometric method has these advantages because it exploits a geometric property that MAP configurations must exhibit. By searching only configurations with this property, our method dramatically reduces the possible search space.

**Figure 4:**
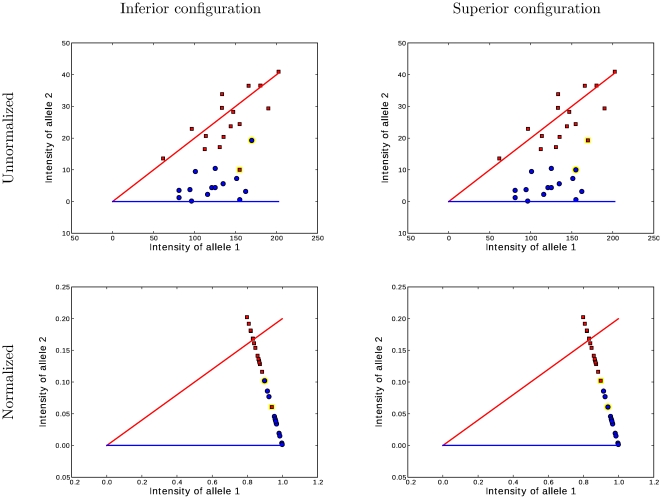
Illustration of a Suboptimal Genotype Configuration. The essential motivation behind the geometric branch and bound is demonstrated. The top figure shows the original data and the bottom figure shows the data after being normalized to 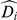 and 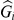 within the likelihood function 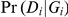. The two figures on the left correspond to a suboptimal genotype configuration. In the figures on the right, a pair of “blue” and “red” points (highlighted) are switched to the opposite class. After swapping the categories, the numbers of individuals with each genotype 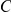 do not change, but the total distance between these two points and their classes decreases. Decreasing this distance increases the likelihood while holding 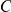 constant. Thus the joint probability 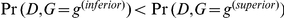. Because the MAP configuration cannot be improved by any such swaps, it must correspond to contiguous groups of class assignments along the normalized axis. Searching only the configurations that result in contiguous class assignments dramatically narrows the search space and makes inference computationally feasible where it wouldn't be with the dynamic programming branch and bound method.

To present our branch and bound method, we first rephrase the problem in a geometric context and then derive a geometric property of optimal configurations (Figure 4). In the likelihood 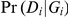, both the data 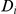 and the theoretical genotypes 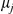 are normalized so that 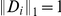 and 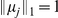. This likelihood is therefore equivalent to 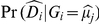. This normalization effectively places the points along the line 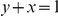. For all 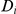 and 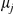, define the operator 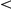 to order them using their normalized values along the line 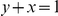 (the direction of the ordering is arbitrary). Similarly, for all 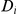 and 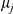 define the distance 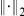 to operate on normalized values of the points on this line. It should be noted that other methods of normalization ( *e.g.* normalizing on a unit circle) will also enable ordering the points in this way and are compatible with this method.

Fix the genotype distribution 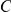. In the joint probability 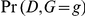 in equation 4, 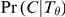 is a constant multiplied by all genotype configurations for which 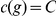. Thus the optimal genotype configuration producing this 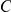 is the one that maximizes the likelihood 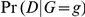. Consider two genotype configurations 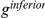 and 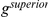 that result in identical genotype distributions 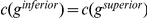. If these configurations are identical except two individuals' genotype assignments, then one configuration must swap the genotype assignments of these individuals (or else the distribution 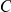 would change). Let these individuals' indices be denoted 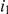 and 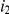 and the possible genotypes be denoted 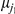 and 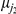. If
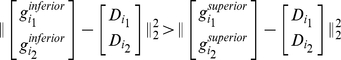
then 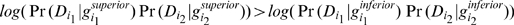 and 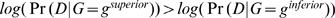. We prove (see Supplement S1) that genotype configurations that do not form contiguous genotype blocks along the line 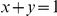 always contain two genotypes that can be swapped to decrease the distance and increase the likelihood; therefore, the optimal genotype configuration consistent with 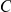 (which cannot be improved without changing 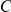) must contain only contiguous blocks of genotype assignments along the line 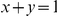.

This approach lets us find the optimal genotype configuration for a given 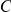 in 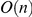 steps by sorting (the sorted order of individuals can be cached and won't vary with the parameters 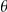 or 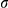). We prove that, for this reason, the optimal genotype configuration can be found by searching possible genotype distributions 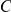 and for each 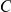 choosing the optimal genotype configuration.

Given a prefix distribution 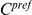, the best genotype configuration prefix 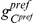 can likewise be trivially found using the sorted order of individuals. In general, we generalize a previous method that performs search on the cardinality of sets rather than on the sets, themselves [32]; our approach generalizes this for the multinomial distribution, rather than a single count. Furthermore, the joint probability of the best genotype configuration consistent with the prefix distribution is bounded above by the product of the multinomial bound, the prefix likelihood, and the best remaining suffix likelihood (more thorough proof shown in Supplement S1):
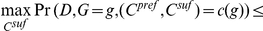


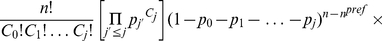


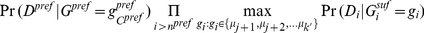
(5)Using this formula, branch and bound can be performed on the tree composed of the search space for the distribution 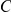; unlike the naive tree and the dynamic programming graph, the tree of all possible distributions has a significantly smaller depth of 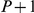, rather than 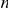. Furthermore, performing branch and bound on this tree is significantly more efficient and can utilize information from a prefix 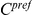 ( *e.g.* using the multinomial and restricting the suffix genotype configurations) to establish a much tighter bound. This method lets us efficiently find the exact MAP 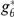 for any 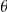 and the overall MAP 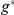.

#### Approximating the Posterior Probability of the MAP Configuration

Given an initial guess at the MAP configuration 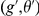 (from the greedy search), it is possible to simultaneously compute the MAP configuration 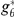 and also approximate the posterior probability of 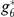. This posterior probability is of great practical utility because it indicates the reliability of the results by quantifying how much better the MAP configuration is compared to all other configurations. In order to approximate the posterior of the MAP, we make two assumptions: first, most of the joint distribution's mass is from the neighborhood nearby the MAP, and second, the posterior distribution of configurations in these neighborhoods behave similar for different values of 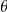. Using these two assumptions, we can approximate the marginal probability as proportional to the joint probability of the MAP:
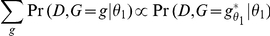


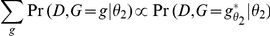
where the constant of proportionality is similar for 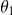 and 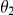.

Therefore, the posterior of a configuration can be approximated:
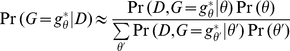
(6)


Denote the greedy genotype configuration for 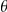 as 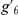 and the greedy configuration with highest posterior 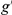. During the branch and bound, it is possible to bound only configurations with joint probabilities so low, omitting them cannot significantly influence the denominator, and hence the overall value of equation 6. To the best of our knowledge, this is the first application of branch and bound to numerical marginalization; in our approach the maximum absolute posterior error (provided as a parameter) determines how conservative the approach must be to bound subtrees when estimating the posterior of the MAP configuration.

Rather than bound any distribution prefix for which all joint probabilities provably inferior to 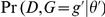, we can only bound distribution prefixes that are substantially inferior. For some 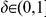, we bound configurations when 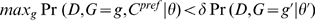 (where the maximum is conservatively estimated using the upper bound from equation 5). Larger values of 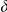 permit more aggressive bounding and smaller values bound more conservatively. We demonstrate (see Supplement S1) that the greatest absolute posterior error 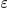 is bounded by the product of 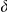 and the total number of parameter configurations queried (not including the MAP): 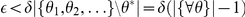. Given 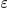, the minimum allowed 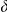 can be found 
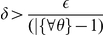
.

#### Approximating Posterior Probabilities for Each Genotype Assignment

It is important to distinguish the configuration posterior (which we approximate above) from posterior estimates that each individual is assigned the correct genotype. SuperMASSA, our implementation of the proposed efficient geometric inference method, also approximates the posteriors for each individual by using the relative likelihood between the MAP genotype and the other possible genotypes for that individual. The user is allowed to set a threshold for this value, and only the individuals with a likelihood ratio exceeding this posterior will be reported (in both figures and output genotype assignments). This approach formalizes heuristics that filter out data points with a total intensity 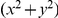 less than some threshold.

Furthermore, it is possible to extend our approach to compute exact posteriors for each genotype assignment. The space searched by branch and bound would be much more complex; however, the MAP genotype configuration computed above would provide the most efficient possible bound. When the MAP has a substantial portion of the probability mass, nearly every subtree will be bounded, resulting in a very efficient runtime.

## Results

### Runtime Improvement with Geometric Branch and Bound

**Figure 5:**
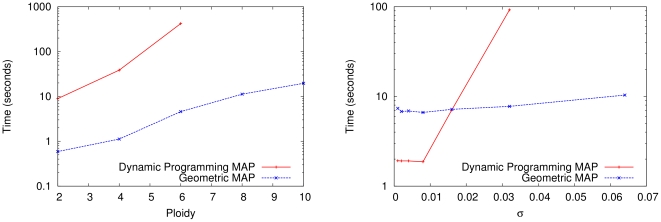
Runtime of Exact MAP Computation with Dynamic Programming and Geometric Branch and Bound. Both methods were run and timed while solving the same MAP inference problem. The y-axis plots the log of the runtime in seconds and the x-axis plots either the ploidy or the parameter 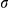. Both methods were implemented in Python and run on sugarcane locus SugSNP225 and timed using user time. Parental data was not used. For this data set, the optimal ploidy and 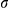 values are 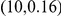. In the figure on the left, 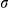 is held constant at 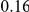 and the ploidy is varied from 2 to 10. In the figure on the right, the ploidy is fixed at 10 and 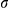 is doubled successively from 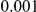 to 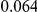. In both figures, the dynamic programming branch and bound series is incomplete because the method was terminated after using more than 3 GB of RAM. In comparison, the geometric branch and bound method never used more than 50 MB. The growing gap between the methods indicates a superpolynomial speedup, especially when larger ploidies and larger values of 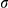 are used. For very low 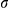 values, the dynamic programming method is sometimes slightly faster due to decreased overhead.

The improved runtime of our geometric branch and bound method relative to the dynamic programming method is a nontrivial change; it makes exact MAP computation feasible where it was not before. In Figure 5, we demonstrate the relationship between the ploidy 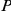, the parameter 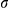 and the runtime of these methods using the SugSNP225 locus. Not only is the geometric method substantially more efficient for more difficult problems (over 100 times faster in some instances), the gap between the two methods grows nonlinearly (as shown by the increasing gap on the log-scale runtimes). Furthermore, the amount of memory used by the dynamic programming method is prohibitively large; in both cases, the dynamic programming runtime series is terminated early for using more than 3 GB of RAM. Most importantly, the dynamic programming time and memory requirements prohibit analysis using the optimal parameters. The optimal ploidy for this locus is 10 and the optimal 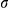 value is 0.16; it is infeasible to run the dynamic programming method for any ploidy greater than four (when 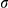 is at its optimal value 0.16) and for any 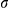 greater than 0.03 when the ploidy is at its optimal value of 10. For this reason, the dynamic programming method could not practically be applied to this data set.

### Inference Results from Potato and Sugarcane Data

For all loci investigated, Table 1 shows the ploidy and number of clusters predicted by both the expert and SuperMASSA. The application of our method provided very good results for the SNPs evaluated, both for potato (diploid and tetraploid) and sugarcane. For potato, SuperMASSA was able to find the correct ploidy level and number of clusters in all cases. For sugarcane the ploidy level was the same for 21 SNPs. For the remaining loci, SuperMASSA predicted similar ploidies for four (differences from 10 to 8 in SugSNP004, 12 to 14 in SugSNP013, and 8 to 6 in SugSNP186 and SugSNP204) and incorrect ploidies (10 to 14 in SugSNP060 and 6 to 14 in SugSNP114). It is important to note that the curated result is not sacrosanct; the exact answer is not known, since the ploidy level is unknown for sugarcane. The number of clusters for sugarcane was the same for 24 SNPs, with only small differences in the remaining. Interestingly, this happened only for loci with different results for ploidy level as well.

**Table 1:** SuperMASSA Results on Potato and Sugarcane Loci.

		PotSNP				SugSNP										
		diploid		Tetraploid												
		016	034	016	034	004	005	013	037	041	045	048	050	060	065	077
Ploidy	Expert	2*	2*	4*	4*	10	6	12	10	8	8	6	10	10	8	6
	SuperMASSA	2	2	4	4	8	6	14	10	8	8	6	10	14	8	6
# Clusters	Expert	3*	3*	5*	5*	2	2	3	2	2	2	2	4	3	2	2
	SuperMASSA	3	3	5	5	2	2	5	2	2	2	2	4	4	2	2

		SugSNP															
		079	082	088	114	117	122	136	151	162	186	201	204	225	235	237	241
Ploidy	Expert	6	8	6	6	10	12	12	6	8	8	10	8	10	8	8	8
	SuperMASSA	6	8	6	14	10	12	12	6	8	6	10	6	10	8	8	8
# Clusters	Expert	2	2	2	2	3	2	2	2	2	2	3	2	4	2	2	2
	SuperMASSA	2	2	2	4	3	2	2	2	2	2	3	2	4	2	2	2

SuperMASSA was run on the potato loci (in both diploid and tetraploid individuals) and on the 27 curated sugarcane loci. The ploidy and number of clusters predicted by an expert are shown with the row label “Expert.” The ploidy predicted by SuperMASSA agreed with the expert on all potato loci and on 21 of the 27 sugarcane loci. The number of clusters predicted by SuperMASSA agreed with the expert on all potato loci and on 24 of the 27 sugarcane loci. The ploidy is known and the number of clusters predicted by [19] is used for each data set, and so the results are marked with an 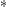 to indicate that expert curation was unnecessary.

Further investigation into the loci where the expert and SuperMASSA disagree revealed that the distributions resulting from the ploidies set by the expert were quite divergent from the theoretical distributions expected for any possible sets of parents. The expert did not analyze these distributions when curating the data, because it was prohibitively time-consuming: the number of possible parents for the considered ploidy range (two to 16) totals 444; enumerating all sets of parents for the 241 considered sugarcane loci would have resulted in 107,004 figures requiring manual analysis.

#### SuperMASSA Output from Selected Potato and Sugarcane Loci

SuperMASSA was run on two potato loci (from both the diploid and tetraploid individuals) and on sugarcane loci using the same parameters. The ploidy range searched was 2 to 16 (only even ploidies were searched) and the 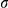 range searched was 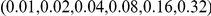. For the sugarcane data, peak heights were used as the measure of intensity ( SuperMASSA has the option of using the peak areas for MassARRAY data). Figures reported were generated automatically without manual editing using the –save_figures option. Parental data (consisting of 12 replicates of each parent) was used for sugarcane loci (results were very similar without using this data).

**Figure 6:**
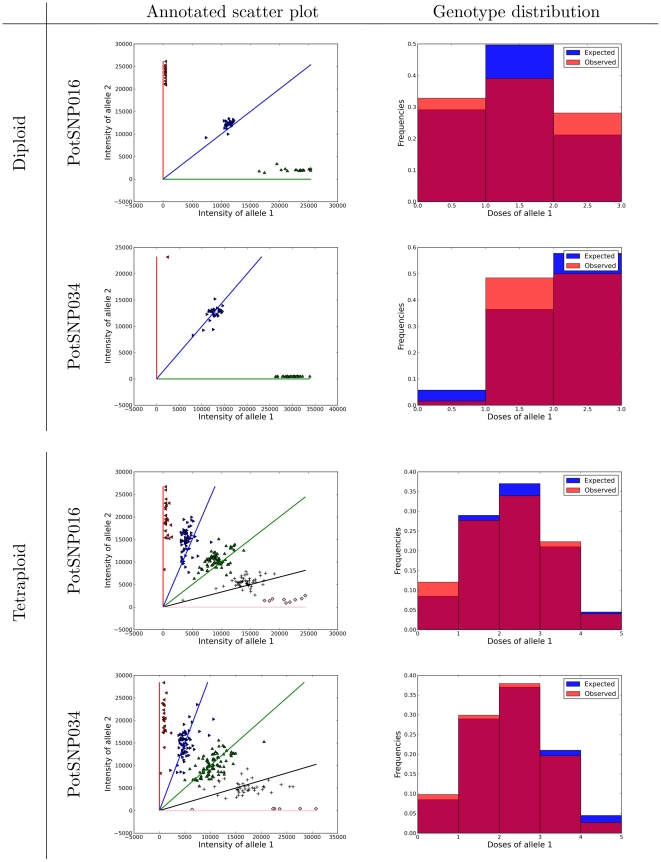
SuperMASSA Output on Potato Loci. For each potato locus (and the ploidy of the species analyzed), we show the output of SuperMASSA (using the –save_figures option). The first column shows the annotated scatter plot and the second column shows the theoretical distribution of genotypes in the population and the distribution of individuals assigned to each genotype. For both loci (in both the diploids and tetraploids), the genotype annotations are extremely close to the predicted angles for each assigned genotype and the genotype distributions are nearly identical to the theoretical distribution in a Hardy-Weinberg population using the MAP estimate for the parameter 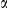.

Figure 6 shows the output from SuperMASSA on potato loci from the diploids and tetraploids. For the diploid potato used as reference by [19], it is easy to see that the results strongly agree with what is expected. First, the observed and estimated proportion of individuals on each class of the distribution are very close to each other. Second, there are 3 clusters corresponding to alleles with 0, 1 or 2 copies. It is also possible to see that there is no skew on the clusters around the expected angles for each cluster (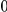, 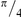 and 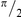). It is important to note that the method was able to deal with clusters containing few individuals. More importantly, the ploidy level was correctly estimated as two. In individuals of the tetraploid potato variety, the results also indicated that the proposed method works well. The estimated ploidy was four, there are five clusters, and the expected and observed proportions under HWE are quite similar. Little skew from the expected angles was observed.

**Figure 7:**
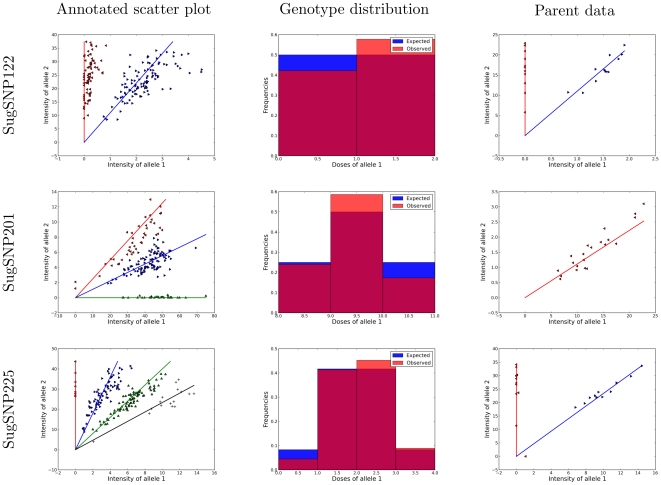
SuperMASSA Output on Sugarcane Loci. For each sugarcane locus, we show the output of SuperMASSA (using the –save_figures option). The first column shows the annotated scatter plot and the second column shows the theoretical distribution of genotypes in the population and the distribution of individuals assigned to each genotype. In all three loci, the MAP configuration simultaneously finds the ploidy, a set of parents with that ploidy, and genotype assignments with tight clusters that produce nearly identical theoretical genotype distributions and genotype distributions.

Figure 7 shows the output from SuperMASSA on three sugarcane loci. For each of these loci, there is a strong agreement between the expected and observed number of individuals in each cluster for an 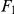. There is no evidence of skew on the annotated scatter plots and individuals were correctly allocated to clusters close to the expected angles for the given ploidy and estimated dosage on parents. Furthermore, the expected angles of these estimated parent dosages closely matched the angles seen in the scatter plot of parent genotype data. The ploidy level was correctly estimated based on what is expected from eye-curation: 12 for SugSNP122, 10 for SugSNP201 and 10 for SugSNP225. The allele dosage in the parents was also estimated as simplex
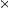
nulliplex, simplex
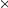
simplex and triplex
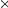
nulliplex, respectively.

## Discussion

These results presented were possible only because our novel approach to inference substantially reduced the search space and permitted much greater utilization of available information ( *e.g.* prior knowledge about rare genotype frequencies) in the branch and bound. We present a geometric interpretation of how our procedure reparameterizes and decreases the size of the search space; however, the key mathematical concept that allowed us to discover the geometric property of optima was due to an exploitation of symmetry. In general, it is possible to condition on outcomes of nodes in a graphical model that perform associative operations (in this instance counting), even though these nodes depend jointly on the state of all predecessor nodes. This is possible by effectively collapsing predecessor configurations that lead to the same outcome. In state-of-the-art software packages for graphical models [33], this type of symmetry may not be exploited to its full potential, and so for our problem, the best runtime for an exact result would have had a worst-case time exponential in the number of individuals. In the future, these special types of dependencies could be identified automatically; it is possible that this type of symmetry is hidden in myriad other problems and could be exploited.

One such straightforward generalization that could be made to our model would use a latent variable to represent the skew of each locus. A prior probability on the skew with a unique mode at zero (no skew) would choose a skewed solution only if it was inferior to all solutions with a skew of zero. Performing inference using a discretization of this latent variable would simply multiply by a constant the runtime of our method. This improvement, though simple, would be quite useful for fluorescence-based genotyping assays, which are sometimes prone to distortion in the relative intensities of each allele.

It is important to note that the method that we present is not exclusively for polyploids; instead, it is a generalized method that is applicable to any ploidy. This is especially important since our method generalizes independent mixture models so that the genotypes of individuals are considered and assigned in concert rather than one at a time. Because of its simple and modular nature, both our model and the inference procedures could be trivially inserted into existing methods. Perhaps even more importantly, the mathematical inference problem we solve is nearly identical to important inference problems proposed for analysis of copy number variation; the platforms that we tested our method on are of great importance for identifying copy number variants. Our method (or components of the model or inference algorithm) could be applied to the relative ratio intensities (due to copy number rather than ploidy) described in [16].

Our approach undoubtedly simplifies the model of meioses in polyploids. However, even when the assumptions of our meiotic model are violated, the anomalous or seemingly contradictory results ( *e.g.* parents with a ploidy different from some or all progeny in an 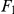), are extremely informative. By using a simple available model of meioses in polyploids, our approach will facilitate the discovery of loci with these anomalous behaviors; identifying and studying examples that violate a simple meiotic model is crucial for furthering our understanding of and developing more accurate models of meiosis in polyploids. A greater understanding of these processes will not only benefit the study of polyploids, it will add insight into the processes involved in cell biology.

### Availability

Our software SuperMASSA is implemented in Python and freely available as an online application at http://statgen.esalq.usp.br/SuperMASSA. The data from the sugarcane loci analyzed are also available at this URL. The potato data analyzed is available in [23].

## Supporting Information

Supplement S1 **Proof that both the dynamic programming branch and bound and geometric branch and bound find the MAP genotype configuration.**
(PDF)
