# Supplementary material for: Efficient Exact Maximum a Posteriori Computation for Bayesian SNP Genotyping in Polyploids
Source: PLoS One. 2012 Feb 17;7(2):e30906. doi: 10.1371/journal.pone.0030906 (PMC3281906; doi:10.1371/journal.pone.0030906)
Supplement: Supplement S1 — Proof that both the dynamic programming branch and bound and geometric branch and bound find the MAP genotype configuration. (PDF) [file pone.0030906.s001.pdf]

Efficient Exact Maximum a Posteriori Computation for Bayesian SNP Genotyping in Polyploids

Oliver Serang[1,2,*], Marcelo Mollinari[3], Antonio Augusto Franco Garcia[3]
1 Department of Neurobiology, Harvard Medical School, Boston, MA United States
2 Department of Pathology, Children's Hospital Boston, Boston, MA, United States
3 Department of Genetics, University of São Paulo/ESALQ, Piracicaba, São Paulo, Brazil
*** E-mail: Oliver.Serang@Childrens.Harvard.edu**

1 Proof of Maximum a Posteriori Genotypes by Searching the Distribution C

Lemma 1 *Given* $\sigma > 0$, $\Pr(D|G)$ *decreases monotonically with* $\|D-G\|_2^2$.

Proof

$$\begin{aligned} log(\Pr(D|G)) &= \sum_i log(\Pr(D_i|G_i)) \\ &= -\sum_i \frac{(D_i - G_i)^2}{2\sigma^2} - \sqrt{2\pi\sigma} \\ &= c_\sigma^{(1)} - c_\sigma^{(2)} \sum_i (D_i - G_i)^2 \\ &= c_\sigma^{(1)} - c_\sigma^{(2)} \|D-G\|_2^2 \end{aligned}$$

where $c_\sigma^{(1)}$ and $c_\sigma^{(2)}$ depend only on σ and $c_\sigma^{(2)} > 0$. ∎

Lemma 2 *Given* $\sigma > 0$ *and* $D_1 < D_2$, *the genotype assigment* $G = g = (g_1, g_2, g_3, g_4, \ldots, g_n)$ *where* $g_1 = \mu_1, g_2 = \mu_0$ *s.t.* $\mu_1 > \mu_0$ *is less likely than genotype assignment* $G = g' = (g'_1, g'_2, g_3, g_4, \ldots, g_n)$ *where* $g'_1 = \mu_0, g'_2 = \mu_1$.

Proof

$$\begin{aligned} \Pr(D|G) &= \prod_i \Pr(D_i|G_i) \\ &= \Pr(D_1|G_1)\Pr(D_2|G_2) \prod_{i:i>2} \Pr(D_i|G_i) \end{aligned}$$

$$\begin{aligned} &\underset{(G_1,G_2)\in\{(g_1,g_2),(g'_1,g'_2)\}}{\text{argmax}} \Pr(D_1|G_1)\Pr(D_2|G_2) \prod_{i:i>2} \Pr(D_i|G_i) \\ = &\underset{(G_1,G_2)\in\{(g_1,g_2),(g'_1,g'_2)\}}{\text{argmax}} \Pr(D_1|G_1)\Pr(D_2|G_2) \end{aligned}$$

because $\prod_{i:i>2} \Pr(D_i|G_i) > 0$.

By Lemma 1, $\Pr(D_1|G_1)\Pr(D_2|G_2)$ decreases monotonically with $\|(D_1, D_2) - (G_1, G_2)\|_2^2$.

$$
\begin{aligned}
\| \begin{bmatrix} D_1 \\ D_2 \end{bmatrix} - \begin{bmatrix} \mu_0 \\ \mu_1 \end{bmatrix} \|_2^2 \quad - \quad \| \begin{bmatrix} D_1 \\ D_2 \end{bmatrix} - \begin{bmatrix} \mu_1 \\ \mu_0 \end{bmatrix} \|_2^2 \\
&= -2[D_1, D_2] \begin{bmatrix} \mu_0 \\ \mu_1 \end{bmatrix} + 2[D_1, D_2] \begin{bmatrix} \mu_1 \\ \mu_0 \end{bmatrix} \\
&= 2[D_1, D_2] \left(\begin{bmatrix} \mu_1 \\ \mu_0 \end{bmatrix} - \begin{bmatrix} \mu_0 \\ \mu_1 \end{bmatrix} \right) \\
&= 2 \left(D_1(\mu_1 - \mu_0) - D_2(\mu_1 - \mu_0) \right) \\
&= 2(\mu_1 - \mu_0)(D_1 - D_2) < 0
\end{aligned}
$$

Therefore, $\Pr(D_1|G_1 = \mu_1) \Pr(D_2|G_2 = \mu_0) < \Pr(D_1|G_1 = \mu_0) \Pr(D_2|G_2 = \mu_1)$ and $\Pr(D|G = g) < \Pr(D|G = g')$ ∎

Lemma 3 *Given the number of individuals with each genotype $C_j = |\{i : \ g_i = \mu_j\}|$, the search space of consistent genotype configurations is $\{g : \ C\} = \{g \in \{\mu'_0, \mu'_1, \ldots \mu'_{k'}\}^n : C\}$ where $\{\mu'_0, \mu'_1, \ldots \mu'_{k'}\} = \{\mu_j : \ j \in \{\ 1, \ldots k\}, C_j > 0\}$.*

Proof

$$C_j = 0 \leftrightarrow \forall i g_i \neq \mu_j$$

Therefore, $\{g : \ C\} = \{\mu_j : \ j \in \{\ 1, \ldots k\}, C_j > 0 : C\}^n$. ∎

Lemma 4 *Given ploidy $P = p$, $\sigma > 0$, data and labels ordered so that $D_1 < D_2 < \ldots < D_n$ and $\mu'_0 < \mu'_1 < \ldots < \mu'_{k'}$, and genotype counts C (where μ'_i and C are defined in Lemma 3), then in the most likely genotype configuration $g^* = (g^*_1, g^*_2, \ldots g^*_n) = \operatorname{argmax}_g \Pr(D|G = g)$, $g^*_1 = \mu'_0$*

Proof By Lemma 3,

$$
\begin{aligned}
& \operatorname*{argmax}_{g \in \{\mu_0, \mu_0, \ldots \mu_P\}^n : C} \Pr(D|G = g) \\
= & \operatorname*{argmax}_{g \in \{\mu'_0, \mu'_1, \ldots \mu'_{k'}\}^n : C} \Pr(D|G = g)
\end{aligned}
$$

$\forall g : \ \ g_1 = \mu'_j \neq \mu'_0$, there must be some $i' > 1$ for which $g_{i'} = \mu'_0$ (because $C_0 > 0$ and the μ_j are unique for a given ploidy). Given that the D_i are sorted in ascending order, then $D_{i'} > D_1$ and $\mu'_0 < \mu'_j$. By Lemma 2, choosing g' such that $g'_1 = \mu_0$ and $g'_{i'} = \mu'_j$ does not change the genotype counts, but increases the probability. Therefore, any configuration with $g_1 \neq \mu'_0$ is suboptimal. Hence by contradiction, in any optimal configuration g^*, $g^*_1 = \mu'_0$. ∎

Theorem 5 *Given ploidy $P = p$, $\sigma > 0$, data and labels ordered so that $D_1 < D_2 < \ldots < D_n$ and $\mu'_0 < \mu'_1 < \ldots < \mu'_{k'}$, and genotype counts C (where μ'_i and C are defined in Lemma 3), then the unique*

most likely genotype configuration is given by

$$\begin{aligned}
g^*_1 &= \mu'_0 \\
g^*_2 &= \mu'_0 \\
&\vdots \\
g^*_{C_0} &= \mu'_0 \\
g^*_{C_0+1} &= \mu'_1 \\
&\vdots \\
g^*_{C_0+C_1} &= \mu'_1 \\
g^*_{C_0+C_1+1} &= \mu'_3 \\
&\vdots \\
g_{C_0+C_1+C_2} &= \mu'_3 \\
&\vdots \\
g^*_{C_0+C_1+C_2+\ldots+C_{k'-1}+1} &= \mu'_{k'-1} \\
&\vdots \\
g^*_{C_0+C_1+C_2+\ldots+C_{k'}} &= \mu'_{k'}
\end{aligned}$$

Proof By Lemma 4 if $g^* = argmax_{g:C} \Pr(D|G = g)$, then $g^*_1 = \mu'_0$. Then $g^* = (g^*_1, {g^{(2)}}^*)$ and $g^* = argmax_{g:C, g^*_1 = \mu'_0} \Pr(D|G = g) = \text{argmax}_{g^{(2)}:C^{(2)}} \Pr(D|G = g^{(2)})$ where $C^{(2)} = (C_0 - 1, C_1, \ldots C_{k'})$. Inductively, this creates a series of maximization problem of the same form. For maximization problem i in this series, the smallest remaining μ'_j for which $C^{(i)}_j > 0$ is assigned to g^*_i. For this reason, μ'_0 is assigned to $g^*_1, \ldots g^*_{C_0}$ because they correspond to the smallest $D_1, \ldots D^*_{C_0}$. After $g^*_1, \ldots g^*_{C_0}$ are assigned, then the new smallest value of μ'_j s.t. $C^{(C_0)}_j > 0$ will be μ'_1; therefore, μ'_1 will be assigned to the next C_1 genotypes $g^*_{C_0+1}, \ldots g_{C_0+C_1}$, μ'_3 will be assigned to the next C_2 genotypes, $g^*_{C_0+C_1+1}, \ldots g_{C_0+C_1+C_2}$, etc. until all genotypes have been filled.

Corollary 6 *Given a distribution prefix $C^{pref} = (C_0, C_1, \ldots C_j)$ with total sum n^{pref}, for all suffixes C^{suf}, the optimal genotype configuration must include the optimal genotype configuration must include the genotype assignments resulting from the subproblem on $C^{pref}, g^{pref}, n^{pref}$ where $g^{pref} = (g_1, g_2, \ldots g_{n^{pref}})$ are in sorted order. Call this prefix configuration $g^{pref}_{C^{pref}}$.*

Proof For any distribution configuration $C = (C^{pref}, C^{suf})$, Theorem 5 defines the optimal genotype configuration by sorting the unassigned individuals after the smallest $C_0, C_1, \ldots$ are assigned. Any genotype configuration that violates this ordering for a smaller problem will necessarily violate for any suffix C^{suf}; therefore, in the optimal configuration, the order must be applied in C^{pref} to achieve optimality.

Theorem 7 *Let the prior on G be uniform (not all configurations will be weighted equally because configurations yielding a more probable distribution C will be weighted more). Given ploidy $P = p$, sigma > 0, and the theoretical distribution T, the genotype configuration that maximizes the posterior is given by $g^* = \{g^*_C : \forall C \Pr(D|G = g^*_C) \Pr(C|T) = f^*\}$, where f^* denotes the maximum value of $\Pr(D|G = g) \Pr(C|T)$ and g^*_C is defined by Theorem 5 for the given genotype counts C.*

Proof Denote the genotype counts for a given configuration as $c(g)$. Let $f(g, c(g)) = \Pr(D|G =$

$g)\Pr(C = c(g)|T)$.

$$\begin{aligned} f^* &= \max_g f(g, c(g)) \\ &= \max_{g,c':c'=c(g)} f(g, c') \\ &= \max_{c':\exists g,c'=c(g)} \max_{g:c'=c(g)} f(g, c') \\ &= \max_{c'} \max_{g:c'=c(g)} f(g, c') \end{aligned}$$

because every considered genotype count is attainable from some genotype configuration.

Theorem 5 states that for a given c', $g^*_{c'}$ attains the unique maximum $\max_{g:c'=c(g)} \Pr(D|G = g)$. For any fixed c', $\Pr(C = c'|T)$ is a postive constant, and so $g^*_{c'}$ also maximizes $f(g, c')$.

Therefore,

$$f^* = \max_{c'} \max_{g:c'=c(g)} f(g, c') = \max_{c'} f(g^*_{c'}, c')$$

If $f(g, c(g)) = f^*$, then g must attain the maximum for that $c(g)$, $\max_{g:c'=c(g)} f(g, c')$. Because $\max_{g:c'=c(g)} f(g, c')$ has a unique optimum $g^*_{c'}$ for any c', then any optimal g^* must be in the set $\{g^*_{c'} : \forall c'\}$ and must attain the maximum f^*. ∎

By Theorem 7, the optimal genotype configuration can be found by searching all C and choosing the g^*_C that maximizes $\Pr(D|G = g^*_C)$. Given that genotype configurations have uniform prior (before being weighted by the distribution $C = c(g)$ that each produces), then the configuration that maximizes $\Pr(D|G = g)$ will maximize $\Pr(G = g|D)$.

2 Branch and Bound

Lemma 8 *The multinomial probability*

$$\binom{n}{C_0}\binom{n - C_0}{C_1}\binom{n - C_0}{C_1}\cdots\binom{n - C_0 - C_1 - \cdots - C_{k-1}}{C_k} p_1^{C_0} p_2^{C_1} \cdots p_k^{C_k}$$

is bounded above by

$$\binom{n}{C_0}\binom{n - C_0}{C_1}\binom{n - C_0}{C_1}\cdots\binom{n - C_0 - C_1 - \cdots - C_{i-1}}{C_i} \times p_1^{C_0} p_2^{C_1} \cdots p_i^{C_i} (1 - p_1 - p_2 - \cdots - p_i)^{n - C_0 - C_1 - \ldots - C_i}$$

for any $i < k$.

Proof $\binom{n}{n'} p^{n'} (1 - p)^{n-n'} \leq 1$ because it defines a single term in the binomial expansion series $(p + 1 - p)^n$ and each term in the series is nonnegative. The value

$$\binom{n}{C_0}\binom{n - C_0}{C_1} p_1^{C_0} p_2^{C_1} (1 - p_2)^{n - C_0 - C_1} \leq \binom{n}{C_0} p_1^{C_0} (1 - p_1)^{n - C_0}$$

because a positive constant $\binom{n}{C_0} p_1^{C_0}$ can be divided out. By induction, extending the series from i to $i+1$ must decrease it; therefore, since $k > i$, the series value must be smaller than the series value for i. ∎

Theorem 9 *Given $T_\theta = (p_0, p_1, \ldots p_P)$ and $C^{pref} = (C_0, C_1, \ldots C_j)$ with $C_0 + C_1 + \ldots + C_j = n^{pref}$, the joint probability of the best genotype configuration compatible with that distribution is bounded by:*

$$\operatorname*{argmax}_g \max_{C^{suf}} \Pr(D, G = g, (C^{pref}, C^{suf}) = c(g)) \leq$$

$$\frac{n!}{C_0! C_1! \ldots C_j!} \left[\prod_{j' \leq j} {p_{j'}}^{C_j} \right] (1 - p_0 - p_1 - \ldots - p_j)^{n - n^{pref}} \times$$

$$\Pr(D^{pref} | G^{pref} = g^{pref}_{C^{pref}}) \prod_{i > n^{pref}} \max_{g_i : g_i \in \{\mu_{j+1}, \mu_{j+2}, \ldots \mu_{k'}\}} \Pr(D_i | G_i^{suf} = g_i)$$

Proof Corollary 6 states that the optimal genotype configuration given C^{pref} is $g^{pref}_{C^{pref}}$. Lemma 8 proves the multinomial bound $\frac{n!}{C_0! C_1! \ldots C_j!} (1 - p_0 - p_1 - \ldots - p_j)^{n - n^{pref}} \geq \Pr((C^{pref}, C^{suf}) | T_\theta)$. Lastly, the greatest suffix likelihood given C^{pref} is the maximum likelihood over all suffixes that can result in C^{pref}. Since $C = (C^{pref}, C^{suf}) = c(g^{pref}) + c(g^{suf})$ and $C^{pref} = c(g^{pref})$, then $c(g^{suf})_{j'} = 0 \; \forall j' \leq j$; therefore, g^{suf} cannot contain any genotypes from $\mu_0, \mu_1, \ldots \mu_{k'}$, and so the maximum likelihood is the maximum likelihood over the remaining genotypes. ∎

3 Approximate Posterior Computation

Theorem 10 *Given approximate posteriors defined as follows:*

$$\Pr(G = g^*_\theta | D) = \frac{\Pr(D, G = g^*_\theta | \theta) \Pr(\theta)}{\sum_{\theta'} \Pr(D, G = g^*_{\theta'} | \theta') \Pr(\theta')}$$

and the following criteria for bounding:

$$max_g \Pr(D, G = g, C^{pref} | \theta) < \delta \Pr(D, G = g' | \theta')$$

Then denote B as the set of θ for which all configurations are eventually bound (and thus do not contribute to the posterior approximation):

$$B = \{\theta : \; \Pr(D, G = g^*_{C^*_\theta}, C^*_\theta, \theta) < \delta \Pr(D, G = g', \theta')\}$$

then the maximum absolute posterior error is $< \delta(|\{\forall \theta\}| - 1)$.

Proof Denote $s_\theta = \Pr(D, G = g^*_\theta | \theta) \Pr(\theta)$ then the posterior for θ can be defined as $\frac{s_\theta}{\sum_{\theta''} s_{\theta''}}$. Denote the denominator in this computation d and the denominator in the approximated computation $d^{(H)} = d - \sum_{\theta' \in H} s_{\theta'}$.

Because θ', by definition, cannot be in B:

$$\begin{aligned}
\frac{d}{d^{(B)}} &< \frac{d^{(B)} + s_{\theta'} \delta |B|}{d^{(B)}} \\
&= 1 + \frac{s_{\theta'} \delta |B|}{d^{(B)}} \\
&< 1 + \frac{s_{\theta'} \delta |B|}{s_{\theta'}} \\
&= 1 + \delta |B|
\end{aligned}$$

$$
\begin{aligned}
\epsilon_\theta &= |\frac{s_\theta}{d} - \frac{s_\theta}{d^{(B)}}| \\
\forall\theta\epsilon_\theta &< |1 - \frac{d}{d^{(B)}}|
\end{aligned}
$$

because $\forall\theta\ \frac{s_\theta}{d} \geq 0$.

Since $\frac{d}{d^{(B)}} > 1$,

$$
\begin{aligned}
|1 - \frac{d}{d^{(B)}}| &= \frac{d}{d^{(B)}} - 1 \\
&< 1 + \delta|B| - 1 \\
&= \delta|B| \\
&\leq \delta(|\{\forall\theta\}| - 1)
\end{aligned}
$$

Because B cannot, by definition, include θ^*. ∎

4 MAP Validity with Replicate Data

Lemma 11 *Given r replicate data points for each individual, the genotype distribution C, and σ, the MAP configuration found by using the mean value of these data points for each individual results in the true MAP configuration.*

Proof Denote the replicate data for individual 1 as $D^{(1)} = (D_1^{(1)}, D_2^{(1)}, \ldots D_r^{(1)})$. The log likelihood of the genotype configuration g is:

$$
\begin{aligned}
& f(\sigma) + \sum_i \sum_k^r \frac{\|D_k^{(i)} - g_i\|_2^2}{\sigma^2} \\
& = f(\sigma) + \frac{1}{\sigma^2} \sum_i \left[\sum_k^r {D^{(i)}}_k^2 \right] + r g_i^2 - 2 \sum_k^r D_k^{(i)} g_i
\end{aligned}
$$

Because $\sum_k^r {D_k^{(i)}}^2$ is a constant that does not depend on θ or g, any g that maximizes the above equation will maximize the following:

$$
\frac{1}{\sigma^2} \sum_i r g_i^2 - 2 \sum_k^r D_k^{(i)} g_i = \frac{1}{\sigma^2} \sum_i g_i^2 - 2 g_i \ mean(D^{(i)})
$$

The equation to maximize without replicate data is:

$$
\frac{r}{\sigma^2} \sum_i D_i^2 + g_i^2 - 2 g_i D_i
$$

For fixed r both functions are different by a constant and thus by using the means of the replicate data, the optimal genotype configuration for C can be reached using Theorem 5. ∎
